# Cognitive Performance Concomitant With Vision Acuity Predicts 13-Year Risk for Mortality

**DOI:** 10.3389/fnagi.2019.00065

**Published:** 2019-03-22

**Authors:** Huan Liao, Zhuoting Zhu, Hongxuan Wang, Xiaoming Rong, Charlotte Aimee Young, Ying Peng

**Affiliations:** ^1^Department of Neurology, Sun Yat-sen Memorial Hospital, Sun Yat-sen University, Guangzhou, China; ^2^Guangdong Provincial Key Laboratory of Malignant Tumor Epigenetics and Gene Regulation, Sun Yat-sen Memorial Hospital, Sun Yat-sen University, Guangzhou, China; ^3^State Key Laboratory of Ophthalmology, Zhongshan Ophthalmic Center, Sun Yat-sen University, Guangzhou, China; ^4^Department of Ophthalmology, University of California, San Francisco, San Francisco, CA, United States

**Keywords:** cognitive performance, vision acuity, joint impact, mortality, NHANES

## Abstract

**Objective:** To assess the joint impact of cognitive performance and visual acuity on mortality over 13-year follow-up in a representative US sample.

**Methods:** Data from National Health and Nutrition Examination Survey (NHANES) participants (≥18 years old) were linked with the death record data of the National Death Index (NDI) with mortality follow-up through December 31, 2011. Cognitive performance was evaluated by the Digit Symbol Substitution Test (DSST) and cognitive performance impairment was defined as the DSST score equal to or less than the median value in the study population. Visual impairment (VI) was defined as presenting visual acuity worse than 20/40 in the better-seeing eye. Risks of all-cause and specific-cause mortality were estimated with Cox proportional hazards models after adjusting for confounders.

**Results:** A total of 2,550 participants 60 years and older from two waves of (NHANES, 1999–2000, 2001–2002) were included in the current analysis. Over a median follow-up period of 9.92 years, 952 (35.2%) died of all causes, of whom 239 (23.1%), 224 (24.0%), and 489 (52.9%) died from cardiovascular disease (CVD), cancer, and non-CVD/non-cancer mortality, respectively. Cognitive performance impairment and VI increased the odds for mortality. Co-presence of VI among cognitive impaired elderly persons predicted nearly a threefold increased risk of all-cause mortality [hazard ratios (HRs), 2.74; 95% confidence interval (CI), 2.02–3.70; *P* < 0.001) and almost a fourfold higher risk of non-CVD/non-cancer mortality (HR, 3.72; 95% CI, 2.30–6.00; *P* < 0.001) compared to having neither impairment.

**Conclusion:** People aged 60 years and over with poorer cognitive performance were at higher risk of long-term mortality, and were especially vulnerable to further mortality when concomitant with VI. It is informative for clinical implication in terms of early preventive interventions.

## Introduction

Life expectancy in industrialized countries has been projected to increase continuously, with most of the projected gains occurring in older ages (Kontis et al., 2017). However, a non-negligible disadvantage to gains in longevity is the increased risk of the impairment of both vision and cognition with age. VI is highly prevalent among aged people, not only leading to morbidity, but imposing an impact on the risk of mortality as well (Zhang et al., 2016; Zhu et al., 2016). It has been estimated that the number of visually impaired people could double by 2050 (Varma et al., 2016). The extended life expectancy and aging population, rising rapidly around the world, have also led to increasing prevalence of dementia (Brookmeyer et al., 2007). Cognitive decline and impairment associated with age have also been proposed to be linked with an increased mortality (Sachs et al., 2011). Hence, well-established identification and management on both visual and cognitive impairment are particularly crucial for improving health in later lives.

Emerging evidence suggests a strong linkage between impairment of vision and cognitive impairment (Rogers and Langa, 2010; Elyashiv et al., 2014; Chen et al., 2017). It has been proposed in large cohort studies that higher rates of cognitive performance impairment exist among aged adults with VI (Elyashiv et al., 2014). In a prospective cohort study, VI has been demonstrated to predict cognitive decline (Lin et al., 2004). Furthermore, several studies have shown improved cognitive performance scores by treating reduced vision via cataract surgery (Tamura et al., 2004; Ishii et al., 2008). Alternatively, severe cognitive performance impairment may raise the risk of incident functional VI, even though the eyes remain structurally healthy (Chen et al., 2017). Although VI and cognitive performance impairment have been associated with mortality separately as mentioned above, much less is known about the joint effects of cognitive impairment and VI on mortality. It has been reported that concomitant VI and cognitive impairment increases mortality in a US study (Liu et al., 2016), while no relation was found in a Japanese study (Mitoku et al., 2016). Notably, in those two studies, VI was assessed by self-reporting and the generalizability of the studies are limited. Furthermore, those two studies did not explore the specific mortality associated with the joint effect of visual and cognitive impairment.

The NHANES is a continuous population-based study, which provides an opportunity to investigate the impact of cognitive performance concomitant with VI on mortality in a nationally representative population of the non-institutionalized US civilian. We hypothesize that not only does cognitive performance impairment and VI independently predicts mortality, but the combination of both also predicts increase mortality.

## Materials and Methods

### Sample and Population

This dataset was extracted from two cycles of NHANES (1999–2000, 2001–2002), which is an ongoing study conducted every 2 years by Centers for Disease Control and Prevention and National Center for Health Statistics (2005). Participants were recruited from the US non-institutionalized civilian population using stratified multistage design, with oversampling of certain subgroups^1^. Participants were invited to a mobile examination center for an extensive examination that included physical examinations, laboratory tests, and questionnaires. All participants were informed about the study and gave their written consent prior to assessments. The study protocols and data collection were conducted according to ethical standards. The research adhered to the guidelines of the Declaration of Helsinki.

### Assessment of Cognitive Performance

The DSST was included in the 1999–2002 cycles of NHANES to assess cognitive performance among participants aged 60 years and older. The DSST is a widely used test with high sensitivity for assessing sustained attention, psychomotor speed, and working memory in epidemiologic and neuropsychologic studies (Wechsler, 2007). Based on codes, participants were required to pair the numbers (1–9) with corresponding symbols. The total number of correctly matched symbols within 2 min was calculated for the DSST points (0–133). Higher correct scores represent better cognitive performance. Since there is no gold standard cutoff for cognitive performance impairment based on the DSST score, we defined cognitive performance impairment as below the median DSST score (DSST value of 40), consistent with methods previously described in other studies (Loprinzi et al., 2017).

### Assessment of Visual Acuity

Presenting visual acuity was evaluated for each eye with participants’ usual distance vision correction with an autorefractor (ARK-760, Nidek Co., Ltd.). Details of methods for visual acuity measurement have been described elsewhere (Ko et al., 2012). Presenting visual acuity worse than 20/40 in the better-seeing eye was used to define VI based on the latest guidelines of the US Preventive Services Task Force (Chou et al., 2016).

### Mortality Data

Mortality data were obtained from the NDI through a probabilistic matching algorithm (Loprinzi and Addoh, 2016). Mortality for NHANES participants (≥18 years old) was followed-up through December 31, 2011. Participants were considered alive when they could not be matched with NDI data. The specific cause of death was coded according to the tenth revision of ICD, Injuries and Causes of death (ICD-10). ICD-10 codes I00-I09, I11, I13, and I20-I51 were used to define the death from heart disease, and codes I60-I69 indicated to the decease from cerebrovascular diseases. The combination codes of deaths from heart disease and cerebrovascular diseases were used to represent deaths from CVD. ICD-10 codes C00-C97 were used to define cancer mortality. Those who were not classified as CVD or cancer-related deaths were considered as death due to non-CVD/non-cancer cause. The follow-up period for each participant was calculated as the time length from the date of the interview to either the date of death or at the end of the follow-up (December 31, 2011), whichever happened first.

### Covariates

Covariates were selected on the basis of established association in previous studies both with exposure (VI or cognitive performance) and outcome (mortality) (Chen et al., 2017). All sociodemographic factors (age, gender, race, education level, marital status, and PIR), health-related behaviors (smoking status and alcohol consumption), and comorbid medical conditions (BMI, diabetes, hypertension, high cholesterol, CRP level, history of CVD, and cancer) were assessed using self-reported questionnaires or laboratory tests.

Ethnicity was classified as Non-Hispanic White, Non-Hispanic Black, Mexican American, and other. Education level was classified as less than high school degree and equal to or more than high school diploma. Marital status (unmarried and other, married/with a partner) was categorized as a two-level covariate. PIR, the indicator of family income, was categorized as below poverty (<1.00) and at or above poverty (≥1.00). Smoking status was defined as two groups: never, former/current smoker. Alcohol consumption was classified as lifetime abstainer or former drinker, current drinker with equal to or less than 3, and current drinker with more than three drinks per week.

Body mass index was calculated as weight in kilograms divided by height in meters squared. Participants were defined as having diabetes if they had self-reported physician diagnosis of diabetes, glycosylated hemoglobin (%) levels of 6.5% or more, or used insulin or the prescription of diabetic agents (Varma et al., 2014). The presence of hypertension was defined by self-reported history of hypertension; or the use of antihypertensive agents; or mean systolic blood pressure ≥140 mm Hg; and/or mean diastolic blood pressure ≥90 mm Hg after three measurements. Individuals were classified as having high cholesterol with serum total cholesterol ≥240 mg/dL or the use of lipid-lowering agent. High level of CRP was defined as CRP 1 mg/dL or over. History of CVD was defined by physician diagnosis of congestive heart failure, coronary heart disease, angina, heart attack, or stroke. History of cancer was based on a previous physician’s diagnosis of cancer.

### Statistical Analysis

We followed the standard NHANES analytic procedures for weighting, taking the complex, stratified design of NHANES into account. Continuous data were presented as means and SEs, and categorical variables were shown as numbers and weighted percentages. We used the design-adjusted one-way analysis of variance and Rao–Scott Pearson χ^2^ for the comparison of continuous and categorical variables, respectively. Plots of survival curves were generated using Kaplan–Meier estimates and the log-rank test was used for comparing the survival distributions among groups. The risks of mortality associated with cognitive performance impairment or VI were estimated using Cox proportional hazards regression models to estimate HRs with 95% CIs. All models were first adjusted for age, gender, ethnicity, and sociodemographic factors (education level, marital status, and income status), and then additionally for BMI, smoking status, drinking status, hypertension, diabetes mellitus, cholesterol level, CRP, history of CVD, and cancer. We also evaluated the joint effect of cognitive performance impairment and VI on mortality by adjusting for multiple covariates. To correct the estimates for non-response, inverse probability weighting was used in the sensitivity analyses. Sensitivity analyses were also performed after Markov chain Monte-Carlo imputations to address the missing data. The proportional-hazards assumption for each variable was tested by generating time-dependent covariates, with *P*-value < 0.05 for the interaction of the variable and a function of survival time regarded as violating the assumption. All variables were found to be valid (*P* > 0.05). All data analyses were performed using STATA (ver. 14.0; StataCorp., College Station, TX, United States). Two-sided *P*-values less than 0.05 were considered to indicate statistical significance.

## Results

A total of 3,234 participants aged 60 years or older participated in NHANES (1999/2002). Among these individuals, 684 were excluded because of missing information on DSST score (522 participants) and missing data on visual acuity (162 participants), leading to a final analytical sample of 2,550 participants. Compared with participants with complete data, participants with incomplete data were older (*P* < 0.001), more likely to be non-Hispanic black (*P* < 0.001) and be unhealthy regarding lifestyle and clinical measures. Other baseline characteristics of subjects excluded and included are shown in Supplementary Table S1. Among the 2,550 participants included in the current analysis, the mean (*SE*) DSST score was 46.9 ± 0.60 points. Table 1 provides basic characteristics of participants overall and by quartile of DSST score. The mean (*SE*) age was 70.6 ± 0.29 years, 56.5% of participants were women and 84.0% non-Hispanic white. Participants with a higher quartile of DSST score were more likely to have better vision, be younger, non-Hispanic white people, well educated, married or with partner, higher income index, current drinker, and healthier in terms of diabetes, hypertension, and history of CVD. There was no significant difference in other characteristics within quartile of DSST score. Supplementary Table S2 illustrates demographic, health-related behaviors, and general health characteristics among participants with and without VI. Older age, non-white ethnicity, lower education level, unmarried status, lower BMI value, hypertension, and history of CVD or cancer were significantly associated with VI.

**Table 1:** Baseline characteristics of participants by digit-symbol substitution test score quintile.

Characteristics	Overall (*N* = 2,550)	DSST score^a^				*P*^b^
		Q1 (*n* = 591)	Q2 (*n* = 630)	Q3 (*n* = 644)	Q4 (*n* = 685)	
VI		
No	2,278 (91.6%)	471 (78.3%)	545 (88.3%)	600 (93.9%)	662 (97.3%)	**<0.001**
Yes	272 (8.4%)	120 (21.7%)	85 (11.7%)	44 (6.1%)	23 (2.7%)	
Age (*SE*), years	70.6 ± 0.29	74.5 ± 0.64	72.7 ± 0.36	71.0 ± 0.36	67.4 ± 0.26	**<0.001**
Gender		
Male	1,257 (43.5%)	312 (42.8%)	337 (48.0%)	325 (45.3%)	283 (39.7%)	0.098
Female	1,293 (56.5%)	279 (57.2%)	293 (52.0%)	319 (54.7%)	402 (60.3%)	
Race						
Non-Hispanic white	1,543 (84.0%)	197 (61.0%)	365 (81.1%)	433 (86.6%)	548 (92.9%)	**<0.001**
Non-Hispanic black	370 (6.5%)	144 (17.6%)	105 (8.0%)	78 (5.1%)	43 (2.2%)	
Mexican American	487 (2.7%)	198 (7.2%)	123 (3.0%)	100 (2.1%)	66 (1.1%)	
Other	150 (6.9%)	52 (14.3%)	37 (7.8%)	33 (6.2%)	28 (3.8%)	
Education		
Less than high school	1,000 (29.0%)	448 (67.0%)	302 (43.0%)	170 (21.9%)	80 (10.5%)	**<0.001**
High school and over	1,549 (71.0%)	142 (33.0%)	328 (57.0%)	474 (78.1%)	605 (89.5%)	
Marital status		
Unmarried and other	911 (35.4%)	248 (49.7%)	259 (43.7%)	227 (35.2%)	177 (24.9%)	**<0.001**
Married/with a partner	1,523 (64.6%)	305 (50.3%)	344 (56.3%)	387 (64.8%)	487 (75.1%)	
PIR		
Below poverty (<1)	340 (12.1%)	175 (36.2%)	85 (12.9%)	58 (9.5%)	22 (3.8%)	**<0.001**
At or above poverty (≥1)	1,926 (87.9%)	341 (63.8%)	492 (87.2%)	521 (90.5%)	572 (96.2%)	
Smoking status		
Never	1,190 (47.0%)	294 (54.1%)	288 (45.5%)	291 (46.3%)	317 (45.6%)	0.091
Former/current	1,355 (53.0%)	296 (45.9%)	341 (54.5%)	352 (53.7%)	366 (54.4%)	
Alcohol consumption		
Lifetime abstainer/former drinker	803 (32.7%)	232 (44.9%)	220 (38.8%)	188 (32.1%)	163 (24.5%)	**<0.001**
Current drinker (≤3 drinks/w)	1,253 (48.9%)	285 (46.7%)	306 (47.4%)	316 (50.7%)	346 (49.3%)	
Current drinker (>3 drinks/w)	434 (18.5%)	55 (8.5%)	89 (13.8%)	123 (17.3%)	167 (26.2%)	
BMI (*SE*), kg/m^2^	28.2 ± 0.14	27.9 ± 0.40	27.9 ± 0.24	28.4 ± 0.25	28.4 ± 0.21	0.412
Diabetes mellitus		
No	1,955 (82.4%)	398 (73.3%)	462 (77.5%)	513 (83.2%)	582 (88.3%)	**<0.001**
Yes	526 (17.6%)	172 (26.7%)	150 (22.5%)	110 (16.8%)	94 (11.7%)	
Hypertension						
No	833 (34.2%)	167 (26.8%)	189 (30.7%)	209 (31.8%)	268 (41.1%)	**<0.001**
Yes	1,659 (65.8%)	403 (73.2%)	428 (69.3%)	421 (68.2%)	407 (58.9%)	
High cholesterol						
No	1,463 (57.6%)	353 (60.9%)	361 (56.7%)	364 (56.5%)	385 (57.5%)	0.656
Yes	978 (42.4%)	199 (39.1%)	246 (43.3%)	253 (43.5%)	280 (42.5%)	
High CRP						
No	2,141 (88.0%)	469 (83.2%)	538 (88.0%)	545 (88.6%)	589 (89.5%)	0.093
Yes	286 (12.0%)	81 (16.8%)	65 (12.0%)	67 (11.4%)	73 (10.5%)	
History of CVD		
No	1,973 (77.1%)	431 (68.4%)	445 (67.0%)	511 (79.3%)	586 (85.0%)	**<0.001**
Yes	577 (22.9%)	160 (31.6%)	185 (33.0%)	133 (20.7%)	99 (15.0%)	
History of cancer		
No	2,068 (78.7%)	510 (80.5%)	500 (77.6%)	513 (78.7%)	545 (78.6%)	0.779
Yes	482 (21.3%)	81 (19.5%)	130 (22.4%)	131 (21.3%)	140 (21.4%)	

*DSST, Digit-Symbol Substitution Test; VI, visual impairment; SE, standard error; BMI, body mass index. Boldface indicates statistical significance. All proportions, means, and SE are weighted estimates of the US population characteristics, taking into account the complex sampling design of the NHANES. ^*a*^Q1–Q4, first quartile–fourth quartile. ^*b*^All *P*-values were calculated using one-way analysis of variance for continuous variables and the design-adjusted Rao–Scott Pearson χ^*2*^ test for categorical variables. ^*a*^Adjusted for age and gender*.

After a median follow-up period of 9.92 years, 952 (35.2%) deaths occurred. Of 952 deaths, 239 (23.1%), 224 (24.0%), and 489 (52.9%) cases were attributed to CVD, cancer, and non-CVD/non-cancer causes, respectively. The comparisons of quartiles of DSST score, VI, and baseline characteristics by mortality status are shown in Table 2. All-cause mortality rates were higher for those participants with a lower quartile of DSST score and those with VI. Age- and gender-adjusted Cox proportional hazards regression models showed covariates, including age, gender, education level, marital status, family income index, smoking status, diabetes, cholesterol level, CRP level, history of CVD, and cancer, were significantly associated with risks of mortality. After adjusting for multiple variables, the Cox models indicated that poorer survival from all-cause, CVD, and non-CVD/non-cancer was associated with cognitive performance impairment (all cause HR, 1.99; 95% CI, 1.60–2.47; CVD HR, 1.72; 95% CI, 1.18–2.51; and non-CVD/non-cancer HR, 2.44; 95% CI, 1.70–3.49) (Table 3). Similarly, VI predicted increased risk of mortality due to all-cause and non-CVD/non-cancer causes in the multifactorial Cox models (all cause HR, 1.51; 95% CI, 1.16–1.96; and non-CVD/non-cancer HR, 1.75; 95% CI, 1.22–2.52).

**Table 2:** All-cause mortality by baseline characteristics.

	Number of
	survived	Number of
	subjects,	died subjects,	Unadjusted
Characteristics	*n* = 1,598 (%)	*n* = 952 (%)	*P*	HR (95% CI)^a^
DSST score		
Quartile 4	546 (45.4%)	139 (19.3%)	**<0.001**	Reference
Quartile 3	425 (28.3%)	219 (24.8%)		**1.37** (**1.11**–**1.69**)
Quartile 2	328 (16.4%)	302 (33.1%)		**2.37** (**1.93**–**2.93**)
Quartile 1	299 (10.0%)	292 (22.8%)		**2.37** (**1.91**–**2.94**)
VI		
No	1,475 (94.3%)	803 (86.8%)	**<0.001**	Reference
Yes	123 (5.7%)	149 (13.2%)		**1.43** (**1.18**–**1.73**)
Age (*SE*), years	68.4 ± 0.23	74.7 ± 0.32	**<0.001**	**1.11** (**1.10**–**1.12**)
Gender		
Male	729 (41.2%)	528 (47.7%)	**0.025**	Reference
Female	869 (58.8%)	424 (52.3%)		**0.66 (0.56**–**0.79)**
Race				
Non-Hispanic white	885 (82.2%)	658 (87.2%)	**0.007**	Reference
Non-Hispanic black	238 (6.6%)	132 (6.3%)		1.22 (0.98–1.52)
Mexican American	361 (3.1%)	126 (1.9%)		0.83 (0.67–1.03)
Other	114 (8.1%)	36 (4.6%)		0.70 (0.48–1.02)
Education		
Less than high school	601 (25.7%)	399 (35.1%)	**<0.001**	Reference
High school and over	997 (74.3%)	552 (64.9%)		**0.77 (0.66**–**0.89)**
Marital status				
Unmarried and other	507 (30.4%)	404 (44.7%)	**<0.001**	Reference
Married/with a partner	1,025 (69.6%)	498 (55.3%)		**0.71 (0.59**–**0.85)**
PIR		
Below poverty (<1)	192 (9.6%)	148 (16.4%)	**0.021**	Reference
At or above poverty (≥1)	1,216 (90.4%)	710 (83.6%)		**0.67 (0.49**–**0.93)**
Smoking status		
Never	787 (50.2%)	403 (41.0%)	**<0.001**	Reference
Former/current	807 (49.8%)	548 (59.0%)		**1.58** (**1.36**–**1.83**)
Alcohol consumption		
Lifetime abstainer/former drinker	503 (32.4%)	300 (33.2%)	0.130	Reference
Current drinker (≤3 drinks/w)	771 (47.8%)	482 (50.9%)		1.11 (0.91–1.35)
Current drinker (>3 drinks/w)	283 (19.9%)	151 (16.0%)		0.89 (0.70–1.13)
BMI (*SE*), kg/m^2^	28.6 ± 0.16	27.5 ± 0.20	**<0.001**	1.00 (0.99–1.01)
Diabetes mellitus		
No	1,247 (84.1%)	708 (79.2%)	**0.015**	Reference
Yes	316 (15.9%)	210 (20.8%)		**1.39** (**1.15**–**1.69**)
Hypertension		
No	569 (37.6%)	264 (28.0%)	**<0.001**	Reference
Yes	992 (62.4%)	667 (72.0%)		1.16 (0.96–1.41)
High cholesterol		
No	900 (55.5%)	563 (61.3%)	**0.022**	Reference
Yes	638 (44.5%)	340 (38.7%)		**0.86 (0.75**–**1.00)**
High CRP		
No	1,370 (89.5%)	771 (85.4%)	**0.003**	Reference
Yes	161 (10.5%)	125 (14.6%)		**1.54** (**1.33**–**1.79**)
History of CVD		
No	1,333 (83.0%)	640 (66.1%)	**<0.001**	Reference
Yes	265 (17.0%)	312 (33.9%)		**1.61** (**1.33**–**1.96**)
History of cancer		
No	1,361 (82.2%)	707 (72.3%)	** < 0.001**	Reference
Yes	237 (17.8%)	245 (27.7%)		**1.30 (1.08**–**1.57)**

*DSST, Digit-Symbol Substitution Test; VI, visual impairment; SE, standard error; BMI, body mass index; CI, confidence interval; HR, hazard ratio. Boldface indicates statistical significance. All-cause mortality was assessed through December 31, 2011. All proportions, means, and SEs are weighted estimates of the US population characteristics, taking into account the complex sampling design of the NHANES. Unadjusted *P*-values were calculated using one-way analysis of variance for continuous variables and the design-adjusted Rao–Scott Pearson χ^*2*^ test for categorical variables*.

**Table 3:** Cox proportional hazards models for all-cause and specific-cause mortality by Digit-Symbol Substitution Test score or VI status.

	Model 1^a^	Model 2^b^
Status	(HR and 95% CI)	(HR and 95% CI)
**Cognitive impairment, Present vs. Absent^c^**		
All-cause mortality	**2.00 (1.65**–**2.42)**	**1.99 (1.60**–**2.47)**
Cardiovascular mortality	**1.90 (1.39**–**2.59)**	**1.72 (1.18**–**2.51)**
Cancer mortality	1.21 (0.76–1.90)	1.38 (0.78–2.45)
Non-cancer/non-cardiovascular	**2.57 (1.90**–**3.47)**	**2.44 (1.70**–**3.49)**
mortality
**VI, Present vs. Absent^c^**		
All-cause mortality	**1.37 (1.07**–**1.73)**	**1.51 (1.16**–**1.96)**
Cardiovascular mortality	1.36 (0.87–2.11)	1.43 (0.80–2.56)
Cancer mortality	0.86 (0.46–1.61)	0.97 (0.46–2.01)
Non-cancer/non-cardiovascular	**1.57 (1.09**–**2.24)**	**1.75 (1.22**–**2.52)**
mortality

*HR, hazard ratio; CI, confidence interval; DSST, Digit-Symbol Substitution Test; VI, visual impairment. Boldface indicates statistical significance. All-cause mortality was assessed through December 31, 2011. ^*a*^Model 1: Adjusted for age, gender, race, education level, marital status, income status. ^*b*^Model 2: Model 1 plus additional adjustments for BMI, smoking status, drinking status, diabetes mellitus, hypertension, cholesterol level, CRP, history of CVD, and cancer. ^*c*^Cognitive impairment defined as DSST score ≤ 40 (median score in population); VI defined as presenting visual acuity worse than 20/40*.

**Figure 1:**
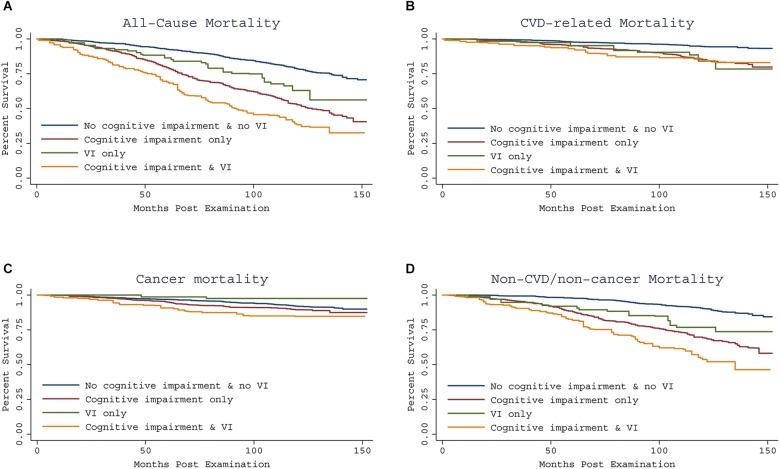
Kaplan-Meier curve showing all-cause and specific-cause mortality by cognitive impairment and concomitant VI, using the 1999–2002 NHANES. All-cause and specific-cause mortality were assessed through December 31, 2011. Compared to having neither impairment, co-presence of VI and cognitive impairment predicted increased risk of all-cause mortality and non-CVD/non-cancer mortality **(A,D)**, but similar risk of CVD and cancer related mortality **(B,C)**.

Kaplan–Meier curves for all-cause and specific-cause mortality by cognitive performance impairment and concomitant VI are shown in Figure 1. Interactions between cognitive performance impairment and VI were assessed, and no significant interaction was found. Table 4 summarizes the stratified analysis regarding the dual impact of cognitive performance impairment with VI on mortality. Considering participants with neither cognitive performance impairment nor VI as the reference group, risk of mortality was increased among those with cognitive performance impairment but without VI (all-cause HR, 1.92; 95% CI, 1.50–2.47; CVD HR, 1.87; 95% CI, 1.22–2.86; cancer HR, 1.28; 95% CI, 0.71–2.30; and non-CVD/non-cancer HR, 2.31; 95% CI, 1.57–3.39). However, participants with only VI did not show higher risk of mortality. Co-presence of VI among cognitively impaired elderly people further increased nearly threefold risk of all-cause mortality (HR, 2.74; 95% CI, 2.02–3.70) and almost fourfold risk of non-CVD/non-cancer mortality (HR, 3.72; 95% CI, 2.30–6.00). We observed similar results to those reported in the main analyses in sensitivity analyses using inverse probability weighting for non-response issue (Supplementary Tables S3, S4). After imputations of missing values, we also obtained similar results (Supplementary Tables S5, S6).

**Table 4:** Cox proportional hazards regression models of all-cause and specific cause mortality by cognitive and visual status.

	Number of	Crude mortality
	events	rate^a^	Model 1^b^	Model 2^c^	*P* for interaction^d^
**All-cause mortality**		
Cognitive and visual status ^e^					0.735
Neither CI nor VI (*n* = 1,262)	331	23.9%	1.00 (reference)	1.00 (reference)	
CI only (*n* = 1,016)	472	51.8%	**1.96** (**1.58**–**2.44**)	**1.92** (**1.50**–**2.47**)	
VI only (*n* = 67)	27	38.7%	1.22 (0.72–2.08)	1.27 (0.77–2.11)	
Both CI and VI (*n* = 205)	122	63.3%	**2.40 (1.83**–**3.16)**	**2.74** (**2.02**–**3.70**)	
**Cardiovascular mortality**		
Cognitive and visual status^e^					0.073
Neither CI nor VI (*n* = 1,262)	77	5.3%	1.00 (reference)	1.00 (reference)	
CI only (*n* = 1,016)	123	12.7%	**2.07 (1.44**–**2.97)**	**1.87** (**1.22**–**2.86**)	
VI only (*n* = 67)	8	14.1%	**2.35 (1.09**–**5.05)**	2.31 (0.98–5.41)	
Both CI and VI (*n* = 205)	31	11.6%	**1.90 (1.11**–**3.25)**	1.87 (0.90–3.88)	
**Cancer mortality**		
Cognitive and visual status^e^					0.069
Neither CI nor VI (*n* = 1,262)	108	8.0%	1.00 (reference)	1.00 (reference)	
CI only (*n* = 1,016)	90	9.3%	1.14 (0.72–1.81)	1.28 (0.71–2.30)	
VI only (*n* = 67)	2	2.1%	0.29 (0.06–1.36)	0.17 (0.02–1.37)	
Both CI and VI (*n* = 205)	24	11.8%	1.26 (0.57–2.80)	1.65 (0.62–4.39)	
**Non-cardiovascular mortality**		
Cognitive and visual status^e^					0.728
Neither CI nor VI (*n* = 1,262)	146	10.7%	1.00 (reference)	1.00 (reference)	
CI only (*n* = 1,016)	259	29.9%	**2.48 (1.79**–**3.44)**	**2.31 (1.57**–**3.39)**	
VI only (*n* = 67)	17	22.5%	1.26 (0.54–2.93)	1.36 (0.62–2.98)	
Both CI and VI (*n* = 205)	67	39.8%	**3.39** (**2.22**–**5.17**)	**3.72** (**2.30**–**6.00**)	

*CI, cognitive impairment; VI, visual impairment. Boldface indicates statistical significance. Values are number of HR (95% CI). All-cause and specific-cause mortality was assessed through December 31, 2011. ^*a*^All proportions are weighted estimates of the US population characteristics, taking into account the complex sampling design of the NHANES. ^*b*^Model 1: Adjusted for age, gender, race, education level, marital status, and income status. ^*c*^Model 2: Model 1 plus additional adjustments for BMI, smoking status, drinking status, diabetes mellitus, hypertension, cholesterol level, CRP, history of CVD, and cancer. ^*d*^Interaction between CI and VI. ^*e*^CI defined as DSST score ≤ 40 (median score in population); VI defined as presenting visual acuity worse than 20/40*.

## Discussion

In a pooled analysis of two nationally representative samples (2,550 US subjects aged 60 years and over), we observed that both cognitive performance impairment and VI independently predicted poorer survival. Additionally, the co-presence of VI among cognitive impaired elderly predicted higher risk of all-cause mortality and of non-CVD/non-cancer mortality compared to having neither impairment.

Visual impairment as a risk factor for cognitive performance (Lin et al., 2004; Fischer et al., 2016; Chen et al., 2017) in the literature was consistent with our result. In addition, the finding that impaired cognitive performance was a significant predictor of mortality was consistent with previous studies (Dewey and Saz, 2001; Perna et al., 2015; Batty et al., 2016; Lee et al., 2018). Moreover, a recent systematic literature review also supports our results (Dewey and Saz, 2001). In terms of VI, our results and those from previous studies suggested that VI increased the risk of all-cause mortality (McCarty et al., 2001;Wang et al., 2001; Khanna et al., 2013; Ng et al., 2018). In a recent meta-analysis, containing 29 prospective cohort studies with 269,839 participants and 67,061 deaths, VI was found to be significantly associated with an increased risk of mortality in participants older than 65 years (Zhang et al., 2016).

In our study, we highlighted the finding that impaired cognitive performance accompanied by VI additionally increased the risk of mortality in our study, which was in line with a study conducted in United States (Liu et al., 2016). On the contrary, in a Japanese study, the joint effect of visual and cognitive impairment did not increase the risk of mortality compared to those without visual/hearing and cognitive impairment, while a significant increase in risk of mortality was observed among individuals with dual impairment (visual and hearing) and cognitive impairment (Mitoku et al., 2016). Taking into account the inclusion of hearing status, self-reported visual function and ethnicity in the Japan study might explain the different results. Of note, even though we observed the significant association between VI and mortality, the association between VI but no impaired cognitive performance and mortality did not reach statistical significance. This might due to the small number of participants in the VI but no impaired cognitive performance group (*n* = 67), which might reduce the efficiency of statistical analysis and limit the ability of detecting the association.

Several possible reasons may account for the joint effect of VI and cognitive performance impairment on mortality. One possible explanation could be that the VI and cognitive impairment contributed additionally to higher possibility of disability (Whitson et al., 2007), which has a close relationship with mortality (Majer et al., 2011). VI not only increases the risks of fractures, falls, and accidents, but also decreases functional independence and ability to perform basic and instrumental activities in daily life (Keller et al., 1999; DiNuzzo et al., 2001), and so these factors could adversely affect mortality. As well, increased risk of falls, accidents, and poor control of underlying diseases due to cognitive impairment could also reduce survival (Dewey and Saz, 2001; Lievre et al., 2008; Muir et al., 2012). Moreover, impaired cognitive performance can predict functional status (Kelman et al., 1994), particularly motor function, which itself is able to affect mortality (McGuire et al., 2006). Another explanation could be underlying vascular diseases. In our analysis, both impaired cognitive performance and VI were associated with hypertension as well as history of CVD, which were significantly related to death. A large number of studies have described a significant relationship between cognitive impairment and CVD (Dregan et al., 2013; Gottesman et al., 2014). In some cases, cognitive impairment is a marker for serious vascular conditions, such as atherosclerosis, cerebrovascular disease, and diastolic hypertension (Gale et al., 1996). Additionally, vision function has also been shown to be associated with cardiovascular-related conditions or risk factors (Bergman et al., 2004; Klein et al., 2014). The underlying vascular dysfunction related to both cognitive and visual health may enhance mortality rate. Further studies are warranted to explore other possible explanations.

Relevant public health implications emerge according to our findings. This could inform clinicians that VI and cognitive performance impairment are likely to have co-occurrence in the elderly and could potentially increase their risks of deaths. Although these conditions are not life threatening, they are recognized as markers for disability and vulnerability. Therefore, neuropsychological tests, combined with vision function tests, could be utilized to perform early identification to maximize the potential impact of preventive interventions. Furthermore, rehabilitation of VI in elders, especially among cognitively impaired persons, may potentially improve long-term survival.

Our study included strengths such as a large population-based elderly cohort, relatively long follow-up duration, standardized objective methods for assessing visual acuity and cognitive performance, access to a wide range of demographic characteristics, health indicators, comorbidities, and access to death records. However, several limitations may limit the interpretation of results. First of all, we could not exclude the residual confounding variables, such as family functioning (Yeh and Liu, 2003; Bambara et al., 2009), which may play a role affecting both VI and cognitive performance, although many confounding factors have been adjusted for. In addition, only baseline measurements of vision function and cognitive performance were employed, and no subsequent functional changes in vision and cognitive performance were examined. Future prospective studies are needed to understand the relationship between changes in cognitive or vision status and survival over time. Thirdly, participants included in the present analysis were younger and healthier regarding lifestyle and clinical measures, which might bias the results. Nevertheless, we used inverse probability weighting model to adjust for non-response and observed similar results. The robustness of our conclusions was again verified after multiple imputation for missing data. Last but not least, cognitive function is a multidimensional construct that cannot be comprehensively evaluated with a single test. However, the DSST is generally thought to be more sensitive than many other measures, especially for milder cognitive impairment. Of note, the DSST exercise relies on visual spatial skills. Although we could not completely mitigate the effect of VI on cognitive performance in the present analysis, the trained interviewer in the NHANES provided safeguards to control this potential confounding effect, including asking participants wear reading glasses when necessary, excluding blind participants and those who were unable to complete a practice session due to visual, physical, or cognitive impairments (NHANES, 1999–2000). Further studies exploring joint effects of cognitive function measured by different tests that are less vision-dependent and visual function on mortality are needed to verify the robustness of our results.

## Conclusion

In conclusion, subjects aged 60 years and older with poorer cognitive performance are at higher risk of decreased long-term survival, and are especially vulnerable to further mortality when VI is presented. This could inform clinicians that early preventive interventions could be exercised to potentially improve long-term survival.

## Data Availability

Data analyzed in current study was from NHANES http://www.cdc.gov/nchs/nhanes.htm/.

## Ethics Statement

This study was carried out in accordance with the recommendations of the National Center for Health Statistics research ethics review board with written informed consent from all subjects. All subjects gave written informed consent in accordance with the Declaration of Helsinki. The protocol was approved by the National Center for Health Statistics research ethics review board.

## Author Contributions

HL, ZZ, and YP study concept and design. HL and ZZ acquisition of data and drafting of the manuscript. HL, ZZ, and XR analysis and interpretation of data. HL, ZZ, HW, XR, CY and YP critical revision of the manuscript for important intellectual content and final approval of the version to be published.

## Conflict of Interest Statement

The authors declare that the research was conducted in the absence of any commercial or financial relationships that could be construed as a potential conflict of interest.

## Footnotes

1 http://www.cdc.gov/nchs/nhanes.htm

## Abbreviations

BMI: body mass index
CIs: confidence intervals
CRP: C-reactive protein
CVD: cardiovascular disease
DSST: Digit Symbol Substitution Test
HRs: hazard ratios
ICD: International Classification of Diseases
NDI: National Death Index
NHANES: National Health and Nutrition Examination Survey
PIR: poverty income ratio
SE: standard errors
VI: visual impairment
