# Supplementary material for: Cognitive Performance Concomitant With Vision Acuity Predicts 13-Year Risk for Mortality
Source: Front Aging Neurosci. 2019 Mar 22;11:65. doi: 10.3389/fnagi.2019.00065 (PMC6439522; doi:10.3389/fnagi.2019.00065)
Supplement: Supplementary file 1 [file Table_1.DOC]

Table S1. Demographic, Health-Related Behaviors and General Health Characteristics of Participants Included and Excluded in the Analysis.

| **Characteristics** | **No. of Excluded Subjects, n = 684 (%)** | **No. of Included Subjects, n = 2,550 (%)** | **P Value** ^a^ |
| --- | --- | --- | --- |
| Age (SE), yrs | 73.8 + 0.38 | 70.6 + 0.29 | **<0.001** |
| Gender |  |  |  |
| Male | 346 (42.6 %) | 1,257 (43.5 %) | 0.729 |
| Female | 338 (57.4 %) | 1,293 (56.5 %) |  |
| Race |  |  |  |
| Non-Hispanic white | 256 (64.2 %) | 1,543 (84.0 %) | **<0.001** |
| Non-Hispanic black | 190 (17.6 %) | 370 (6.5 %) |  |
| Mexican American | 187 (5.2 %) | 487 (2.7 %) |  |
| Other | 51 (13.0 %) | 150 (6.9 %) |  |
| Education |  |  |  |
| Less than high school | 436 (48.6 %) | 1,000 (29.0 %) | **<0.001** |
| High school and over | 248 (51.4 %) | 1,549 (71.0 %) |  |
| Marital status |  |  |  |
| Unmarried and other | 327 (49.9 %) | 911 (35.4 %) | **<0.001** |
| Married/with a partner | 323 (50.1 %) | 1,523 (64.6 %) |  |
| Poverty income ratio (PIR) |  |  |  |
| Below poverty (<1) | 152 (21.8 %) | 340 (12.1 %) | **0.010** |
| At or above poverty (>1) | 394 (78.2 %) | 1,926 (87.9 %) |  |
| Smoking status |  |  |  |
| Never | 343 (50.3 %) | 1,190 (47.0 %) | 0.181 |
| Former/Current | 339 (49.7 %) | 1,355 (53.0 %) |  |
| Alcohol consumption |  |  |  |
| Lifetime abstainer/former drinker | 195 (37.8 %) | 803 (32.7 %) | **0.020** |
| Current drinker  (< 3 drinks/w) | 296 (51.0 %) | 1,253 (48.9 %) |  |
| Current drinker  (> 3 drinks/w) | 59 (11.1 %) | 434 (18.5 %) |  |
| BMI (SE), kg/m^2^ | 28.6 + 0.36 | 28.2 + 0.14 | 0.344 |
| Diabetes mellitus |  |  |  |
| No | 417 (71.7 %) | 1,955 (82.4 %) | **0.001** |
| Yes | 207 (28.3 %) | 526 (17.6 %) |  |
| Hypertension |  |  |  |
| No | 165 (27.7 %) | 833 (34.2 %) | **0.011** |
| Yes | 436 (72.3 %) | 1,659 (65.8 %) |  |
| High cholesterol |  |  |  |
| No | 375 (63.3 %) | 1,463 (57.6 %) | **0.007** |
| Yes | 215 (36.7 %) | 978 (42.4 %) |  |
| High C-reactive protein |  |  |  |
| No | 475 (80.8 %) | 2,141 (88.0 %) | **0.002** |
| Yes | 113 (19.2 %) | 286 (12.0 %) |  |
| History of cardiovascular disease |  |  |  |
| No | 476 (67.9 %) | 1,973 (77.1 %) | **0.004** |
| Yes | 208 (32.1 %) | 577 (22.9 %) |  |
| History of cancer |  |  |  |
| No | 577 (78.6 %) | 2,068 (78.7 %) | 0.971 |
| Yes | 107 (21.4 %) | 482 (21.3 %) |  |
| Abbreviations: SE, standard error; BMI, body mass index. Boldface indicates statistical significance.  All proportions are weighted estimates of the US population characteristics, taking into account the complex sampling design of the National Health and Nutrition Examination Survey.  ^a^ P values were calculated using one-way analysis of variance for continuous variables and the design-adjusted Rao-Scott Pearson χ^2^ test for categorical variables. | | | |

Table S2. Demographic, Health-Related Behaviors and General Health Characteristics of Participants With and Without Visual Impairment.

| **Characteristics** | **No. Subjects without VI, n = 2,278 (%)** | **No. Subjects with VI,**  **n = 272 (%)** | **P Value** ^a^ |
| --- | --- | --- | --- |
| Age (SE), yrs | 70.2 + 0.28 | 75.0 + 0.69 | **<0.001** |
| Gender |  |  |  |
| Male | 1,126 (43.7) | 131 (41.5) | 0.59 |
| Female | 1,152 (56.3) | 141 (58.5) |  |
| Race |  |  |  |
| Non-Hispanic white | 1,398 (85.1) | 145 (71.5) | **0.03** |
| Non-Hispanic black | 329 (6.4) | 41 (8.1) |  |
| Mexican American | 430 (2.6) | 57 (3.8) |  |
| Other | 121 (6.0) | 29 (16.7) |  |
| Education |  |  |  |
| Less than high school | 865 (27.9) | 135 (41.0) | **<0.001** |
| High school and over | 1,413 (72.1) | 136 (59.0) |  |
| Marital status |  |  |  |
| Unmarried and other | 790 (34.3) | 121 (47.3) | **0.03** |
| Married/with a partner | 1,384 (65.7) | 139 (52.7) |  |
| Poverty income ratio (PIR) |  |  |  |
| Below poverty (<1) | 282 (11.1) | 58 (22.1) | 0.09 |
| At or above poverty (>1) | 1,739 (88.9) | 187 (77.9) |  |
| Smoking status |  |  |  |
| Never | 1,051 (46.5) | 139 (52.9) | 0.17 |
| Former/Current | 1,223 (53.5) | 132 (47.1) |  |
| Alcohol consumption |  |  |  |
| Lifetime abstainer/former drinker | 706 (32.1) | 97 (38.9) | 0.06 |
| Current drinker  (< 3 drinks/w) | 1,123 (48.9) | 130 (48.8) |  |
| Current drinker  (> 3 drinks/w) | 401 (19.0) | 33 (12.3) |  |
| BMI (SE), kg/m^2^ | 28.3 + 0.14 | 27.1 + 0.43 | **0.009** |
| Diabetes mellitus |  |  |  |
| No | 1,755 (83.0) | 200 (76.0) | 0.10 |
| Yes | 456 (17.0) | 70 (24.0) |  |
| Hypertension |  |  |  |
| No | 763 (35.0) | 70 (26.1) | **0.04** |
| Yes | 1,468 (65.0) | 191 (73.9) |  |
| High cholesterol |  |  |  |
| No | 1,298 (57.5) | 165 (58.1) | 0.87 |
| Yes | 879 (42.5) | 99 (41.9) |  |
| High C-reactive protein |  |  |  |
| No | 1,920 (88.4) | 221 (84.2) | 0.27 |
| Yes | 247 (11.6) | 39 (15.8) |  |
| History of cardiovascular disease |  |  |  |
| No | 1,769 (77.5) | 204 (72.1) | **0.05** |
| Yes | 509 (22.5) | 68 (27.9) |  |
| History of cancer |  |  |  |
| No | 1,853 (79.2) | 215 (73.0) | **0.03** |
| Yes | 425 (20.8) | 57 (27.0) |  |
| Abbreviations: VI, visual impairment; SE, standard error; BMI, body mass index. Boldface indicates statistical significance.  All proportions are weighted estimates of the US population characteristics, taking into account the complex sampling design of the National Health and Nutrition Examination Survey.  ^a^ P values were calculated using one-way analysis of variance for continuous variables and the design-adjusted Rao-Scott Pearson χ^2^ test for categorical variables. | | | |

Table S3. Cox Proportional Hazards Models for All-Cause and Specific-Cause Mortality by Digit-Symbol Substitution Test Score or Visual Impairment Status Using Inverse Probability Weighting.

| **Status** | **Model 1** ^a^  **(HR and 95% CI)** | **Model 2** ^b^  **(HR and 95% CI)** |
| --- | --- | --- |
| **Cognitive impairment, Present vs Absent** ^c^ |  |  |
| All-cause mortality | **2.09 (1.71-2.56)** | **1.95 (1.58-2.41)** |
| Cardiovascular mortality | **2.04 (1.44-2.88)** | **1.69 (1.17-2.44)** |
| Cancer mortality | 1.22 (0.68-2.19) | 1.33 (0.75-2.39) |
| Non-cancer/non-cardiovascular mortality | **2.64 (1.86-3.74)** | **2.41 (1.69-3.44)** |
| **VI, Present vs Absent** ^c^ |  |  |
| All-cause mortality | **1.47 (1.14-1.89)** | **1.49 (1.16-1.93)** |
| Cardiovascular mortality | 1.39 (0.80-2.43) | 1.42 (0.81-2.48) |
| Cancer mortality | 0.88 (0.43-1.82) | 0.95 (0.46-1.96) |
| Non-cancer/non-cardiovascular mortality | **1.71 (1.22-2.40)** | **1.73 (1.22-2.46)** |
| Abbreviations: HR, hazard ratio; CI, confidence interval; DSST, Digit-Symbol Substitution Test; VI, visual impairment. Boldface indicates statistical significance.  All-cause mortality was assessed through December 31, 2011.  ^a^ Model 1: Adjusted for age, gender, race, education level, marital status, income status.  ^b^ Model 2: Model 1 plus additional adjustments for BMI, smoking status, drinking status, diabetes mellitus, hypertension, cholesterol level, C-reactive protein, history of cardiovascular disease and cancer.  ^c^ Cognitive impairment defined as DSST score < 40 (median score in population); VI defined as presenting visual acuity worse than 20/40. | | |

Table S4. Cox Proportional Hazards Regression Models of All-Cause and Specific Cause Mortality by Cognitive and Visual Status Using Inverse Probability Weighting.

|  | **Model 1** ^a^  **(HR and 95% CI)** | **Model 2** ^b^  **(HR and 95% CI)** | **P for Interaction** ^c^ |
| --- | --- | --- | --- |
| **All-Cause Mortality** | | | |
| Cognitive and visual status ^d^ |  |  | 0.792 |
| Neither CI nor VI (n = 1262) | 1.00 (reference) | 1.00 (reference) |  |
| CI only (n = 1016) | **2.04 (1.61-2.58)** | **1.89 (1.49-2.41)** |  |
| VI only (n = 67) | 1.29 (0.73-2.28) | 1.29 (0.78-2.12) |  |
| Both CI and VI (n = 205) | **2.73 (2.08-3.60)** | **2.65 (1.98-3.56)** |  |
| **Cardiovascular Mortality** | | | |
| Cognitive and visual status ^d^ |  |  | 0.074 |
| Neither CI nor VI (n = 1262) | 1.00 (reference) | 1.00 (reference) |  |
| CI only (n = 1016) | **2.22 (1.50-3.29)** | **1.83 (1.20-2.78)** |  |
| VI only (n = 67) | 2.38 (0.98-5.77) | 2.29 (0.99-5.30) |  |
| Both CI and VI (n = 205) | **2.06 (1.05-4.02)** | 1.85 (0.93-3.69) |  |
| **Cancer mortality** | | | |
| Cognitive and visual status ^d^ |  |  | 0.070 |
| Neither CI nor VI (n = 1262) | 1.00 (reference) | 1.00 (reference) |  |
| CI only (n = 1016) | 1.13 (0.63-2.04) | 1.24 (0.69-2.24) |  |
| VI only (n = 67) | 0.15 (0.02-1.22) | 0.17 (0.02-1.35) |  |
| Both CI and VI (n = 205) | 1.39 (0.52-3.71) | 1.56 (0.59-4.13) |  |
| **Non-cardiovascular Mortality** | | | |
| Cognitive and visual status ^d^ |  |  | 0.792 |
| Neither CI nor VI (n = 1262) | 1.00 (reference) | 1.00 (reference) |  |
| CI only (n = 1016) | **2.54 (1.73-3.71)** | **2.29 (1.57-3.34)** |  |
| VI only (n = 67) | 1.44 (0.63-3.27) | 1.40 (0.65-3.01) |  |
| Both CI and VI (n = 205) | **3.84 (2.49-5.91)** | **3.63 (2.28-5.79)** |  |
| Abbreviations: CI, cognitive impairment; VI, visual impairment.  Boldface indicates statistical significance. Values are number of hazard ratio (95% confidence interval).  All-cause and specific-cause mortality was assessed through December 31, 2011.  ^a^ Model 1: Adjusted for age, gender, race, education level, marital status, income status.  ^b^ Model 2: Model 1 plus additional adjustments for BMI, smoking status, drinking status, diabetes mellitus, hypertension, cholesterol level, C-reactive protein, history of cardiovascular disease and cancer.  ^c^ Interaction between CI and VI.  ^d^ CI defined as DSST score < 40 (median score in population); VI defined as presenting visual acuity worse than 20/40. | | | |

Table S5. Cox Proportional Hazards Models for All-Cause and Specific-Cause Mortality by Digit-Symbol Substitution Test Score or Visual Impairment Status After Multiple Imputation of Missing Data.

| **Status** | **Model 1** ^a^  **(HR and 95% CI)** | **Model 2** ^b^  **(HR and 95% CI)** |
| --- | --- | --- |
| **Cognitive impairment, Present vs Absent** ^c^ |  |  |
| All-cause mortality | **1.96 (1.65-2.33)** | **1.85 (1.55-2.20)** |
| Cardiovascular mortality | **1.92 (1.37-2.69)** | **1.63 (1.14-2.33)** |
| Cancer mortality | 1.21 (0.79-1.83) | 1.25 (0.84-1.85) |
| Non-cancer/non-cardiovascular mortality | **2.48 (1.88-3.26)** | **2.32 (1.74-3.09)** |
| **VI, Present vs Absent** ^c^ |  |  |
| All-cause mortality | **1.45 (1.19-1.78)** | **1.47 (1.22-1.79)** |
| Cardiovascular mortality | 1.40 (0.90-2.18) | 1.47 (0.95-2.27) |
| Cancer mortality | 1.07 (0.62-1.86) | 1.11 (0.65-1.92) |
| Non-cancer/non-cardiovascular mortality | **1.63 (1.17-2.28)** | **1.65 (1.21-2.26)** |
| Abbreviations: HR, hazard ratio; CI, confidence interval; DSST, Digit-Symbol Substitution Test; VI, visual impairment. Boldface indicates statistical significance.  All-cause mortality was assessed through December 31, 2011.  ^a^ Model 1: Adjusted for age, gender, race, education level, marital status, income status.  ^b^ Model 2: Model 1 plus additional adjustments for BMI, smoking status, drinking status, diabetes mellitus, hypertension, cholesterol level, C-reactive protein, history of cardiovascular disease and cancer.  ^c^ Cognitive impairment defined as DSST score < 40 (median score in population); VI defined as presenting visual acuity worse than 20/40. | | |

Table S6. Cox Proportional Hazards Regression Models of All-Cause and Specific Cause Mortality by Cognitive and Visual Status After Multiple Imputation of Missing Data.

|  | **Model 1** ^a^  **(HR and 95% CI)** | **Model 2** ^b^  **(HR and 95% CI)** | **P for Interaction** ^c^ |
| --- | --- | --- | --- |
| **All-Cause Mortality** | | | |
| Cognitive and visual status ^d^ |  |  | 0.901 |
| Neither CI nor VI (n = 1262) | 1.00 (reference) | 1.00 (reference) |  |
| CI only (n = 1016) | **1.93 (1.59-2.35)** | **1.81 (1.50-2.19)** |  |
| VI only (n = 67) | 1.40 (0.91-2.15) | 1.39 (0.93-2.08) |  |
| Both CI and VI (n = 205) | **2.50 (1.99-3.13)** | **2.44 (1.92-3.10)** |  |
| **Cardiovascular Mortality** | | | |
| Cognitive and visual status ^d^ |  |  | 0.206 |
| Neither CI nor VI (n = 1262) | 1.00 (reference) | 1.00 (reference) |  |
| CI only (n = 1016) | **2.05 (1.40-3.01)** | **1.71 (1.14-2.55)** |  |
| VI only (n = 67) | **2.24 (1.01-4.94)** | 2.03 (0.97-4.27) |  |
| Both CI and VI (n = 205) | **2.06 (1.20-3.55)** | **1.99 (1.13-3.52)** |  |
| **Cancer mortality** | | | |
| Cognitive and visual status ^d^ |  |  | 0.052 |
| Neither CI nor VI (n = 1262) | 1.00 (reference) | 1.00 (reference) |  |
| CI only (n = 1016) | 1.10 (0.70-1.72) | 1.14 (0.74-1.75) |  |
| VI only (n = 67) | 0.25 (0.05-1.22) | 0.29 (0.06-1.32) |  |
| Both CI and VI (n = 205) | 1.60 (0.86-2.98) | 1.64 (0.90-2.98) |  |
| **Non-cardiovascular Mortality** | | | |
| Cognitive and visual status ^d^ |  |  | 0.560 |
| Neither CI nor VI (n = 1262) | 1.00 (reference) | 1.00 (reference) |  |
| CI only (n = 1016) | **2.48 (1.83-3.35)** | **2.30 (1.69-3.12)** |  |
| VI only (n = 67) | 1.77 (0.94-3.34) | 1.74 (0.93-3.24) |  |
| Both CI and VI (n = 205) | **3.33 (2.28-4.88)** | **3.24 (2.18-4.81)** |  |
| Abbreviations: CI, cognitive impairment; VI, visual impairment.  Boldface indicates statistical significance. Values are number of hazard ratio (95% confidence interval).  All-cause and specific-cause mortality was assessed through December 31, 2011.  ^a^ Model 1: Adjusted for age, gender, race, education level, marital status, income status.  ^b^ Model 2: Model 1 plus additional adjustments for BMI, smoking status, drinking status, diabetes mellitus, hypertension, cholesterol level, C-reactive protein, history of cardiovascular disease and cancer.  ^c^ Interaction between CI and VI.  ^d^ CI defined as DSST score < 40 (median score in population); VI defined as presenting visual acuity worse than 20/40. | | | |
